# Developments in Mass Spectrometry for Glycosaminoglycan Analysis: A Review

**DOI:** 10.1074/mcp.R120.002267

**Published:** 2021-01-06

**Authors:** Lauren E. Pepi, Patience Sanderson, Morgan Stickney, I. Jonathan Amster

**Affiliations:** Department of Chemistry, University of Georgia, Athens, Georgia, USA

**Keywords:** glycosaminoglycan, carbohydrate, mass spectrometry, tandem mass spectrometry, ATIII, antithrombin III, CID, collision-induced dissociation, CS, chondroitin sulfate, CZE, capillary zone electrophoresis, DMS, differential mobility spectrometry, dp, degrees of polymerization, DS, dermatan sulfate, EDD, electron detachment dissociation, EID, electron-induced dissociation, ETD, electron transfer dissociation, FT-ICR MS, Fourier Transform ion cyclotron resonance mass spectrometer, GAGs, glycosaminoglycans, gated-TIMS, gated-trapped ion mobility spectrometry, HA, hyaluronic acid, HCD, higher-energy collisional dissociation, HILIC, hydrophilic interaction liquid chromatography, Hp, heparin, HS, heparan sulfate, ILM, ionic liquid matrices, IMS, ion mobility spectrometry, IRMPD, infrared multiphoton dissociation, KS, keratan sulfate, LMWH, low-molecular-weight heparin, MS/MS, tandem mass spectrometry, MSI, mass spectrometry imaging, NETD, negative electron transfer dissociation, PG, proteoglycan, ROS, reactive oxygen species, RPIP, reverse-phase ion pairing, SAX, strong anion exchange, SEC, size-exclusion chromatography, UVPD, ultraviolet photodissociation

## Abstract

This review covers recent developments in glycosaminoglycan (GAG) analysis *via* mass spectrometry (MS). GAGs participate in a variety of biological functions, including cellular communication, wound healing, and anticoagulation, and are important targets for structural characterization. GAGs exhibit a diverse range of structural features due to the variety of *O*- and *N*-sulfation modifications and uronic acid C-5 epimerization that can occur, making their analysis a challenging target. Mass spectrometry approaches to the structure assignment of GAGs have been widely investigated, and new methodologies remain the subject of development. Advances in sample preparation, tandem MS techniques (MS/MS), online separations, and automated analysis software have advanced the field of GAG analysis. These recent developments have led to remarkable improvements in the precision and time efficiency for the structural characterization of GAGs.

## Highlights

• Online separation with tandem mass spectrometry can sequence glycosaminoglycans.
• Electronic excitation increases GAG sequence coverage over collisional activation.
• This approach is useful for analysis of biological samples and pharmaceuticals.

## Graphical Abstract

**Figure:**
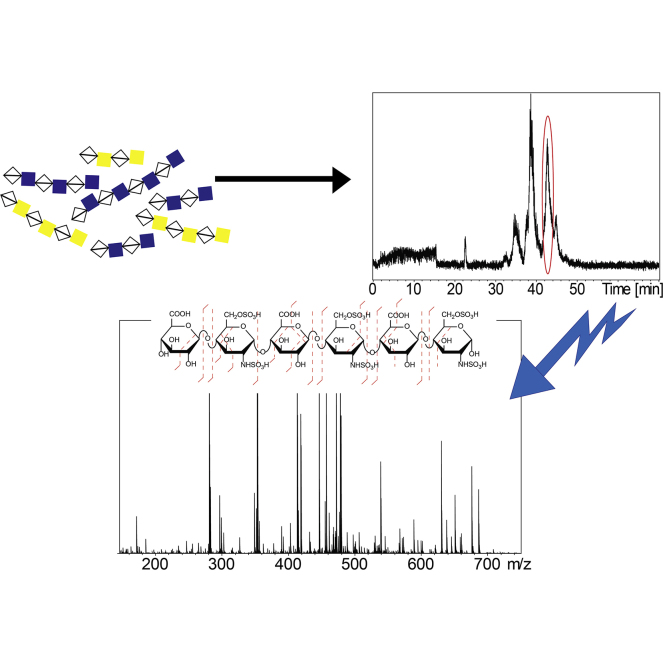

## In Brief

Glycosaminoglycans (GAGs) participate in a variety of biological functions and have a multitude of medicinal properties. Due to their non template driven biosynthesis, GAGs are produced as nonuniform complex mixtures. Mass spectrometry paired with on-line separation techniques has been utilized to determine the composition of these complex mixtures. Advances in tandem mass spectrometry have also made determining sequence information such as sulfation location and C-5 epimerization possible. This review covers recent developments in the analysis of GAGs using mass spectrometry.

## Overview

The structural diversity of glycosaminoglycans (GAGs) makes them challenging targets for analysis. Mass spectrometry (MS) has played an important role in this endeavor, due to its high sensitivity, specificity for discerning subtle differences in structure, and its capability to examine complex mixtures. Proteoglycans (PGs) consist of a core protein along with one or more covalently bound GAG chains (1). The biological function of the PG is typically determined by the GAG component. GAGs are primarily found on the surface of cells or in the extracellular matrix (2). GAGs are classified into four main groups: heparin/heparan sulfate (Hp/HS), chondroitin sulfate/dermatan sulfate (CS/DS), keratan sulfate (KS), and hyaluronic acid (HA) (2). Hp/HS and CS/DS participate in a number of biological processes, and their analysis is the focus of this review. GAGs are long, linear polysaccharides with repeating disaccharide units. Hp/HS and CS/DS are composed of an *N*-acetyl amino sugar and an uronic acid. The first biosynthesis step, chain elongation, produces a uniform repeating polymer of an *N*-acetyl amino sugar (GlcNAc for Hp/HS and GalNAc for CS/DS) and glucuronic acid. The chains are subsequently modified by deacetylases, sulfotransferases, and epimerases to produce highly complex and heterogeneous structures. Sulfo-modified GAGs are negatively charged and highly polar molecules. Due to the complex nature and the biological relevance of Hp/HS and CS/DS, these GAG families have been the focus of considerable research into the development of new MS approaches to analysis.

HA is an unsulfated GAG composed of repeating disaccharide units of *N*-acetylgalactosamine (GalNAc) and glucuronic acid (GlcA) joined by alternating β(1,4) and β(1,3) linkage (2). In contrast to Hp/HS and CS/DS, HA is homogeneous compound unmodified by sulfotransferases or by epimerases. HA is distributed in the neural, connective, and epithelial tissues, with an estimated 15 g of HA in an adult human body. HA can weigh as much as 100 to 10,000 kDa, making it quite large (3). Recently, HA has been a component used in dermal fillers and has been a desired ingredient in many face creams and treatments due to its chemical–physical properties, biodegradability, biocompatibility, and versatility (4, 5). However, due to the homogenous structure, there has not been a significant need to characterize HA using mass spectrometry. KS is made of repeating disaccharide units of galactose (Gal) and GlcNAc joined by alternating β(1,4) and β(1,3) linkage (2). The disaccharide building blocks of KS can be unsulfated, monosulfated, or disulfated. KS is primarily found in the cartilage, cornea, and bone. It has been shown to participate in the development and healing of the central nervous system (6). Though KS is sulfated, it can only be modified at the 6-*O* position on either the Gal or GlcNAc residue, making its structure less complex than Hp/HS or CS/DS, and has not been the subject of much development activity (7, 8).

**Fig. 1:**
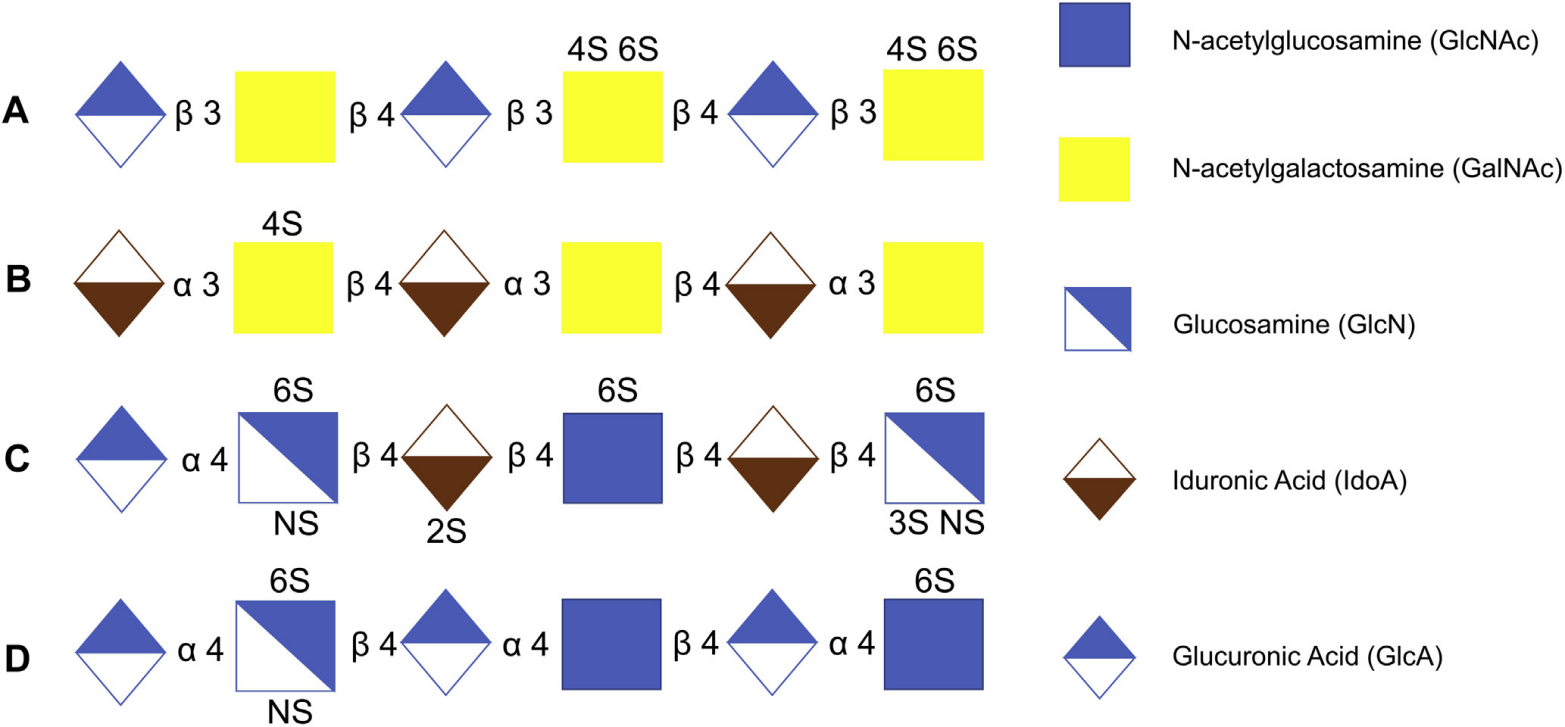
**Example Hp/HS and CS/DS chains.***A*, Chondroitin sulfate chain, *B*, dermatan sulfate chain, *C*, heparan sulfate chain with both IdoA and GlcA and 2-O sulfation, *D*, heparan sulfate chain with GlcA.

CS occurs in a variety of locations within mammals, including extracellular matrix such as connective tissue and cartilage, tethered to proteins on the cell surface, and also as secreted proteoglycans. It is widely used as a treatment for osteoarthritis and cataracts, as it has anti-inflammatory and pain-reducing properties (9, 10). CS is upregulated in the extracellular matrix of scar tissue and perineuronal nets, making it a useful treatment following neural injury (10). CS can be as large as 100,000 kDa (11). CS has a disaccharide backbone composed of GalNAc and GlcA joined by an alternating β(1,4) and β(1,3) linkage, respectively (2, 12). Chondroitin sulfate is polymerized into chains that can be hundreds of residues long and is usually composed of hybrid structures containing more than one type of chondroitin disaccharide unit. There are three principal types of chondroitin sulfate, CS-A, CS-B (also known as dermatan sulfate), and CS-C. CS-A and CS-B are predominantly sulfated at the 4-*O* position of the GalNAc, whereas CS-C has 6-*O* sulfated GalNAc subunits (2). An example of CS and DS is shown in Figure 1, *A* and*B*. CS-B/DS is composed of repeating disaccharide units containing GalNAc and iduronic acid (IdoA), which differs from GlcA only in C-5 stereochemistry (2). Dermatan sulfates are the primary GAGs in the dermis and are responsible for binding proteins involved in modulation of a broad range of physiological processes. Other patterns of modification, including those with two sulfo-groups per disaccharide, have been reported; CS-D has 2-*O* sulfation on the uronic acid and 6-*O* sulfation on the GalNAc, and CS-E has 4-*O* and 6-*O* sulfation on the GalNAc (13).

Hp and HS are structurally the most complex members of the GAG polysaccharides (14). One of the main functions of Hp is anticoagulation and prevention of vein thrombosis and pulmonary embolism. Hp is produced in mast cells and has more sulfates per hexosamine than HS (15). HS is produced by most mammalian cells and located on cell surfaces and in the extracellular matrix (16). Hp can weigh up to ∼14 kDa, whereas HS can weigh up to ∼75 kDa (17). Hp/HS are composed of uronic acid and *N*-acetyl glucosamine repeating disaccharide subunits (16) and joined by alternating α(1,4) and β(1,4) linkage (2). Example heparan sulfate structures are illustrated in Figure 1, *C* and *D*. The uronic acid of the repeating disaccharide unit can be either L-IdoA or D-GlcA, both of which can be 2-*O* sulfated; however, this sulfation pattern is predominantly seen on IdoA (IdoA2S) (15, 18). The D-(GlcN) can be *N*-sulfated (GlcNS) or *N*-acetylated (GlcNAc), both of which could have 6-*O* sulfation, and the GlcNS can also be 3-*O* sulfated (12, 15, 16, 19, 20). Despite an understanding of some of the biological roles GAGs possess, there is still room for development in understanding their structure–function relationship. The complexity of the biosynthesis of GAGs creates complex mixtures and heterogeneous structures, creating a need for the structural characterization of GAGs.

## Analytical Challenge

There is considerable interest in determining the structures of GAGs and relating these to their biological activity. Past research has shown the importance of GAG structure in relation to function, specifically when related to protein binding (19, 21, 22). However, GAG structural analysis remains a significant analytical challenge (23, 24). The biosynthesis of GAGs is a nontemplate-driven, enzymatic process. GAG biosynthesis starts with a homogeneous copolymer that undergoes extensive modification by deacetylase, sulfotransferases, and epimerase. This process results in nonuniform glycan chains with varying degrees of acetylation and sulfation and produces complex mixtures of biological GAGs (2). Additionally, GAGs are generally available only in small quantities and cannot be overexpressed or amplified like other biopolymers, specifically proteins and nucleic acids (25). This combined with their high molecular weight limits the applicability of tools such as nuclear magnetic resonance (NMR) or X-Ray diffraction (26, 27, 28). For these reasons, the development of mass spectrometry methods for GAG analysis has attracted significant research effort. GAGs have two features that impact the MS methodologies that are applied, specifically their anionic nature and the fragility of their sulfo-modifications. MS methodology developments have greatly improved in the last decade as a result of advances in online separations, ion activation techniques, and software for automated analysis of complex MS and MS/MS data, as will be presented below.

## Sample Preparation for MS Analysis

In nature, GAGs generally occur as PGs, in which the glycan chain is covalently attached to the serine residue of a protein. Samples are usually prepared for MS analysis by detaching the glycan from the protein, either by beta-elimination or by complete digestion of the protein to leave a single serine residue at the reducing end of the glycan chain (23, 29, 30, 31, 32). Hp/HS and CS/DS chains are covalently bound to serine residues in core proteins by a four-residue oligosaccharide linker with xylose at the reducing end (HS or CS)-(1,4)-GlcA-(1,3)-Gal-(1,3)-Gal-(1,4)-Xyl-Ser (2). The biosynthesis of both of these classes of GAGs results in heterogeneous mixtures that can be hundreds of residues long. To facilitate their mass spectrometry analysis, these long chains are usually depolymerized to smaller oligomers, although there are published accounts of the mass spectrometry analysis of full-length glycans from two proteoglycans with relatively short chains (30, 31, 32). GAGs can be depolymerized enzymatically by either lyases or hydrolases (Table 1) (29, 33). Lyases catalyze an elimination reaction that abstracts a proton at the C-5 position of the uronic acid in concert with β-elimination of the 4-*O* glycosidic bond, which results in an unsaturated C-4, C-5 bond within the terminal residue, producing a Δ-uronic acid at the nonreducing end of the cleavage site, eliminating the C-5 stereochemistry in this residue (34). In contrast, hydrolases yield conventional uronic acids at the nonreducing end of the cleavage site, preserving the uronic acid stereochemistry. For both lyases and hydrolases, the leaving group abstracts a hydrogen from a solvent molecule to yield a hydroxyl group at its reducing end (34). There are important implications in the interpretation of MS/MS data when a Δ-uronic acid is present, as will be discussed below.

**Table 1:** List of depolymerization methods

Depolymerization method	Pros	Cons
Chondroitinase ABC	• Cleaves IdoA and GlcA	
Chondroitinase B	• Cleaves IdoA	• Does not cleave GlcA
Heparinase I	• Cleaves highly sulfated chains	• Does not cleave sparsely sulfated domains
Heparinase II	• Cleaves highly and sparsely sulfated chains	
Heparinase III	• Cleaves sparsely sulfated chains	• Does not cleave highly sulfated domains
Heparanase Bp	• Cleaves at nonreducing end of sparsely sulfated hexosamine	• Highly sulfated GAGs are resistant
Reactive oxygen species (ROS)	• Depolymerize GAGs resistant to enzymatic digestion	• Cleaves in nonselective manner
Nitrous oxide (NO)	• Works on both sulfated and unsulfated amino sugars • Uronic acid left intact	• Amino sugars become anydromannose

The lyase reactions are highly specific to the family of GAG and to the pattern of modification near the site of cleavage. For CS/DS depolymerization, chondroitinase ABC will accept IdoA or GlcA for degradation, while chondroitinase B is specific for dermatan sulfate and only accepts IdoA (35). For Hp/HS depolymerization, heparinase I cleaves highly sulfated chains, heparinase III cleaves less sulfated chains, and heparinase II cleaves domains of both high and low sulfation. The use of heparinase I, II, and III together can produce a near-complete depolymerization of Hp/HS chains to disaccharides, useful for compositional analysis (35, 36, 37). Incomplete depolymerization is used to generate GAG chains in the dp4–dp20 (degrees of polymerization) range, with retention of the pattern of modification over these short stretches. Heparanase Bp, which is a β-glucuronidase derived from *Burkholderia pseudomallei*, has shown enzymatic activity on a number of GAGs (38). Heparanase Bp was examined using defined heparan sulfate oligomers and biosynthesized intermediates. It was determined that the enzyme cuts the reducing end of GlcA residues and the nonreducing end of sparsely sulfated hexosamine residues (GlcNAc/GlcNS/GlcNAc6S). It was found that highly sulfated polysaccharides showed resistance to heparanase Bp (39).

Depolymerization can also be performed chemically by using reactive oxygen species (ROS) (40, 41, 42). Fenton chemistry reagents, cupric acetate and hydrogen peroxide, are mixed to create hydroxide radicals. These radicals interact with GAGs to cleave glycosidic bonds in a nonselective manner. ROS depolymerization is useful to interrogate GAGs that are not responsive to the enzymatic approaches described above. Li *et al.* (43) utilized radical depolymerization for contaminant identification in low-molecule-weight heparin (LMWH) mixtures. The contaminant components, oversulfated CS oligomers, were resistant to enzymatic digestion due to their structural modifications but were susceptible to ROS depolymerization. Combining this technique with separation and MS/MS, the structure of the GAG contamination species was confirmed. Depolymerization can also be achieved using nitrous oxide (NO), which produces HNO_2_ as the active species that degrades heparin and heparan sulfate. Depolymerization using NO leads to anhydromannose formation at the reducing end of the resulting oligomer, as a result of the sulfated or free amine of a GlcN(S) residue losing its amine group during the elimination of a glycosidic bond (44, 45). The uronic acid moieties are left intact.

Synthetically produced GAG chains provide useful standards for methods development. These can be produced using chemical synthesis or by chemoenzymatic approaches. Chemical techniques rely on organic reactions for *de novo* synthesis of oligosaccharides from monosaccharide building blocks (46). The Boons group proposed a modular synthesis approach for the development of an HS standards library (47). This approach uses disaccharide building blocks resembling different disaccharide motifs found in HS and then assembling these by a parallel combinatorial manner into larger structures. This method depends on the ability to produce the appropriate mono- and disaccharide building blocks and the selective removal of protecting groups in the correct sequence during a multistep synthesis (47). Enzymatic synthesis has the advantage of higher yield compared with chemical synthesis (48). Chemoenzymatic techniques rely on bacterial fermentation of the polysaccharide backbone and use sulfotransferases and epimerases to employ postpolymerization modifications (48, 49). Chemoenzymatic synthesis uses genetically engineered sulfotransferases that provide control over the site of modification. However, with these engineered enzymes, mainly highly sulfated IdoA-GlcN repeating units are formed (48). Chemoenzymatic techniques are less time-consuming than traditional chemical synthesis and can provide higher yields of product. On the other hand, there is more control of sites of modification by using chemical synthesis.

## Mass Spectrometry

### Electrospray Ionization (ESI)

ESI is the standard approach for analyzing GAG samples *via* mass spectrometry. Negative ion mode is typically employed, as the carboxyl groups and sulfate modifications present in GAGs make them highly anionic. GAGs have high ionization efficiency in negative ion mode, and the process can be tuned to be soft enough to avoid loss of labile sulfate modifications (50). ESI of GAGs typically produces multiple charge states and alkali ion heterogeneity (Na^+^/H^+^ exchange), which can either be exploited for controlling ion activation or be suppressed by addition of formic acid or diethylamine to produce a clearer spectrum (51, 52).

### Matrix-Assisted Laser Desorption Ionization (MALDI)

**Fig. 2:**
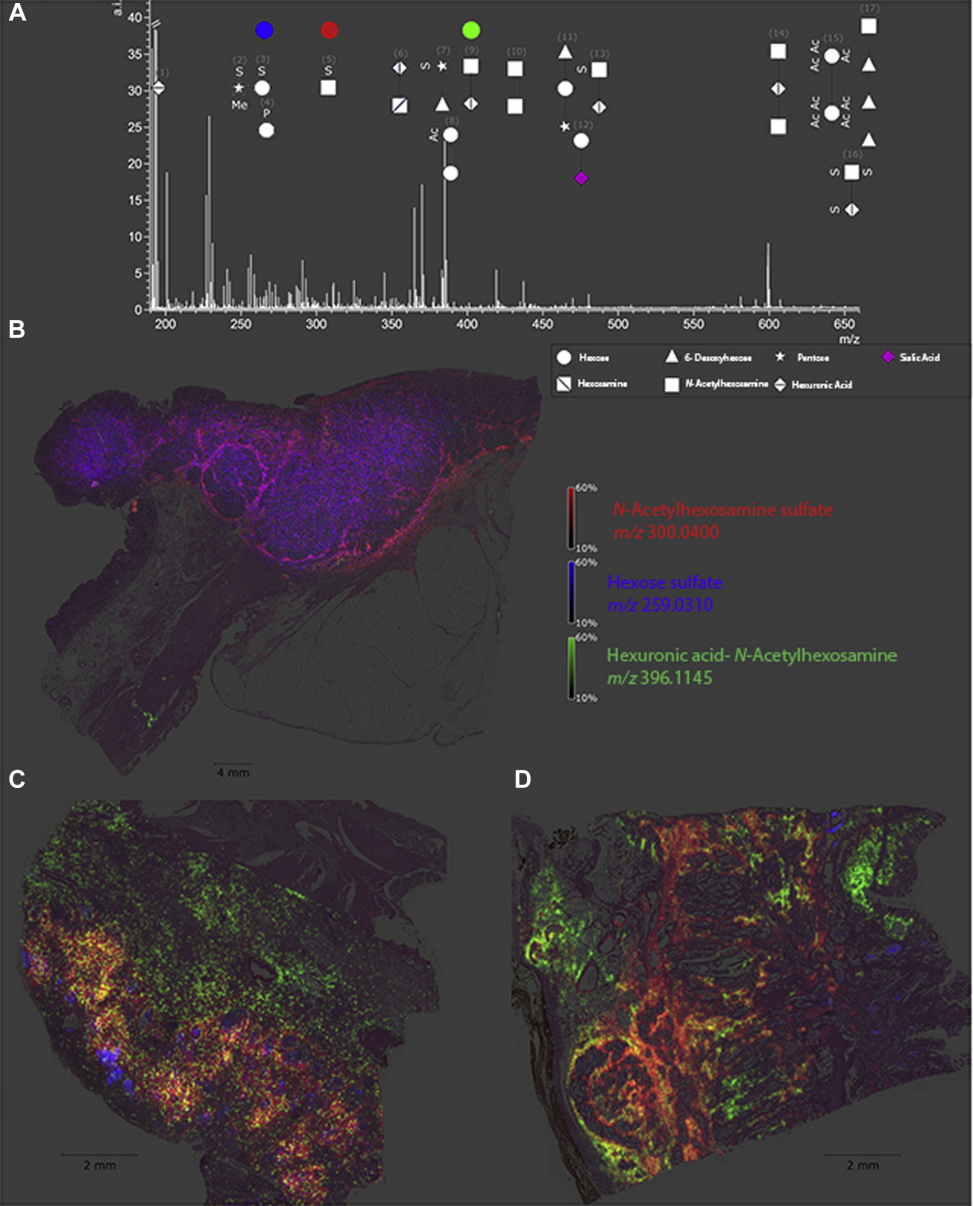
**Detectable native glycan fragments and whole gastric cancer tissue section ion map.***A*, detectable glycan fragments were in the mass range of 190 to 660 m/z and are shown as symbols with numbers. *B*–*D*, ion map of N-acetylhexosamine sulphate, hexose sulfate, and hexuronic acid N-acetylhexosamine in whole tissue sections from a gastric cancer patient. Every tissue section corresponds to an individual patient and highlights altered specific distribution of each glycan fragment. Reprinted with permission from reference (52). Copyright 2017 Oncotarget.

MALDI is another widely used ionization method for biopolymer analysis and is particularly applicable for peptides and proteins as well as their glycoconjugates. The application of MALDI to GAGs is challenging due to their highly anionic nature and the thermal lability of sulfo-modifications. GAGs typically have low ionization efficiency with MALDI in negative ion mode and will readily undergo neutral loss by laser activation, principally by sulfate decomposition. In positive ion mode, ionization efficiency is poor, and the protonated sulfate half-esters are particularly susceptible to neutral loss (53). GAGs can be derivatized or bound to basic peptides to ionize better in positive ion mode (54, 55). Ionic liquid matrices (ILM) and alkali metal counter ions have been shown to increase the ionization efficiency and to modestly improve sulfate stability of GAGs for MALDI in both positive and negative ion modes (56, 57, 58, 59). It has been shown that ILM-MALDI combined with CID can be used to generate structurally informative fragmentation of highly sulfated GAGs (60). MALDI can be paired with separation techniques such as thin layer chromatography (TLC) and gel electrophoresis for improved selectivity in biological samples (61, 62). Recently MALDI mass spectrometry imaging (MALDI-MSI) has identified GAG fragments as biomarkers linked to various diseased tissue samples, including pulmonary fibrosis and gastric cancer tissues as shown in Figure 2 (63, 64).

### Composition Analysis

**Fig. 3:**
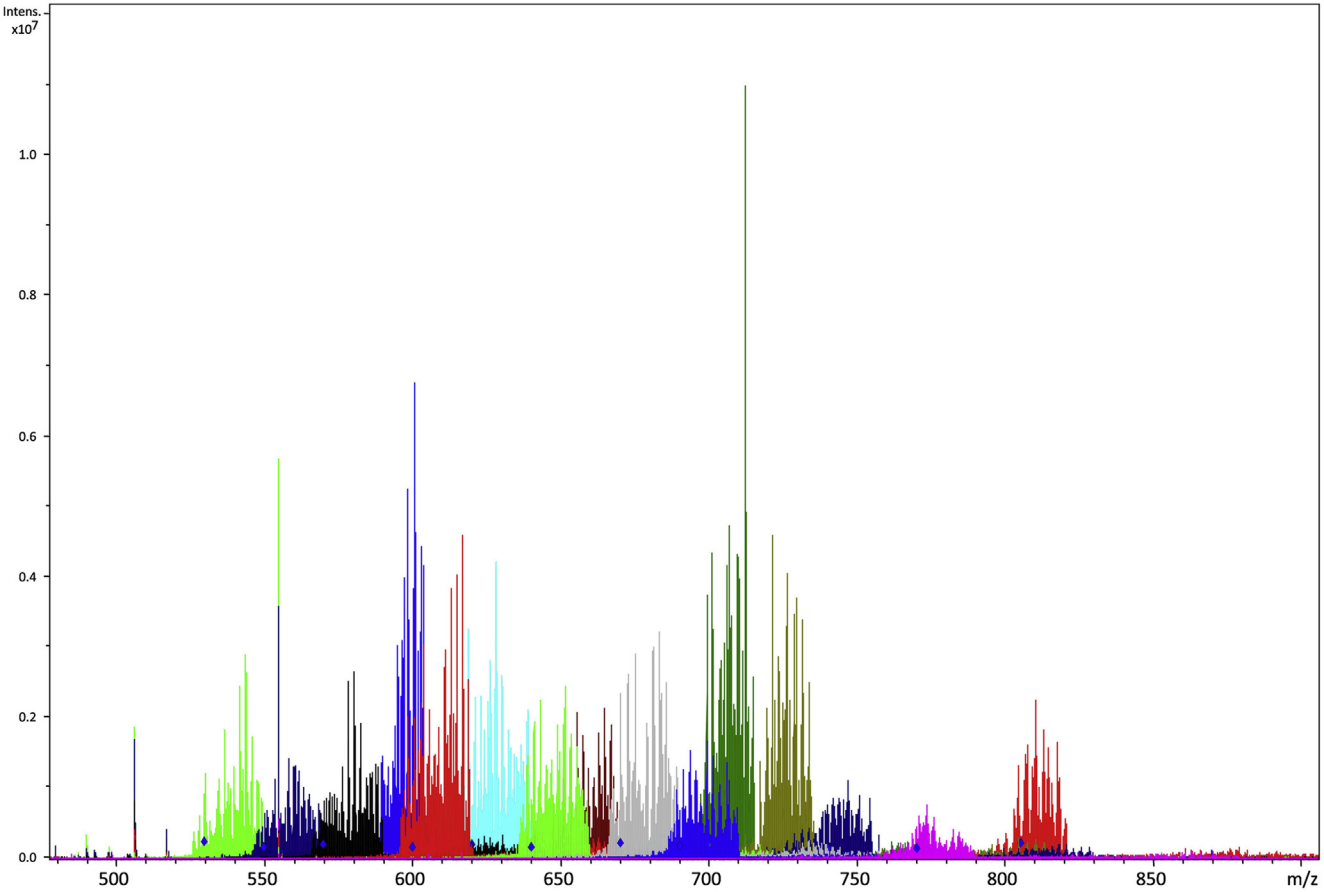
**Improvement in S/N in the FTICR mass spectrum of the bikunin CS mixture achieved by combining mass spectra acquires over narrow overlapping m/z regions.** Reprinted with permission from reference (15). Copyright 2008 American Chemical Society.

Composition analysis is typically the first step in GAG analysis and can be useful for some basic and general information. Accurate mass measurement by MS provides the means to assign chain length (dp) and the type and number of modifications present in a GAG oligomer. Composition analysis can be paired with disaccharide analysis to assign general modification motifs for GAG species (65, 66). General changes in GAG composition have been linked to many medical conditions and developmental biology (67, 68, 69, 70). One challenge in assigning composition based on accurate mass measurement is heterogeneity from sodium/hydrogen exchange. Molecules with a number of ionizable sites, such as GAGs or nucleic acids, are susceptible to replacement of acidic protons by alkali cations. This can produce a broad distribution of molecular species. When convoluted with a distribution of compositions and charge states, this can give rise to complex mass spectra, as seen in Figure 3 for a mixture of full-length CS glycans from bikunin (23). Spreading the molecular ion over a number of alkali exchange states reduces the intensity of the peaks and makes the assignment of composition more difficult. Desalting the sample with a spin filter and adding dilute formic acid or diethylamine to the electrospray solvent can significantly decrease the degree of cation exchange, making the signals stronger by reducing the heterogeneity of the molecular ion (71, 72). High degrees of alkali exchange can result in an added degree of difficulty for analyzing GAGs chromatographically. Ion suppression is often utilized for online chromatographic analysis of GAGs. The ion suppressor removes alkali and ammonium ions from the mobile phase, improving the signal strength (73).

### GAG Sequencing

**Fig. 4:**
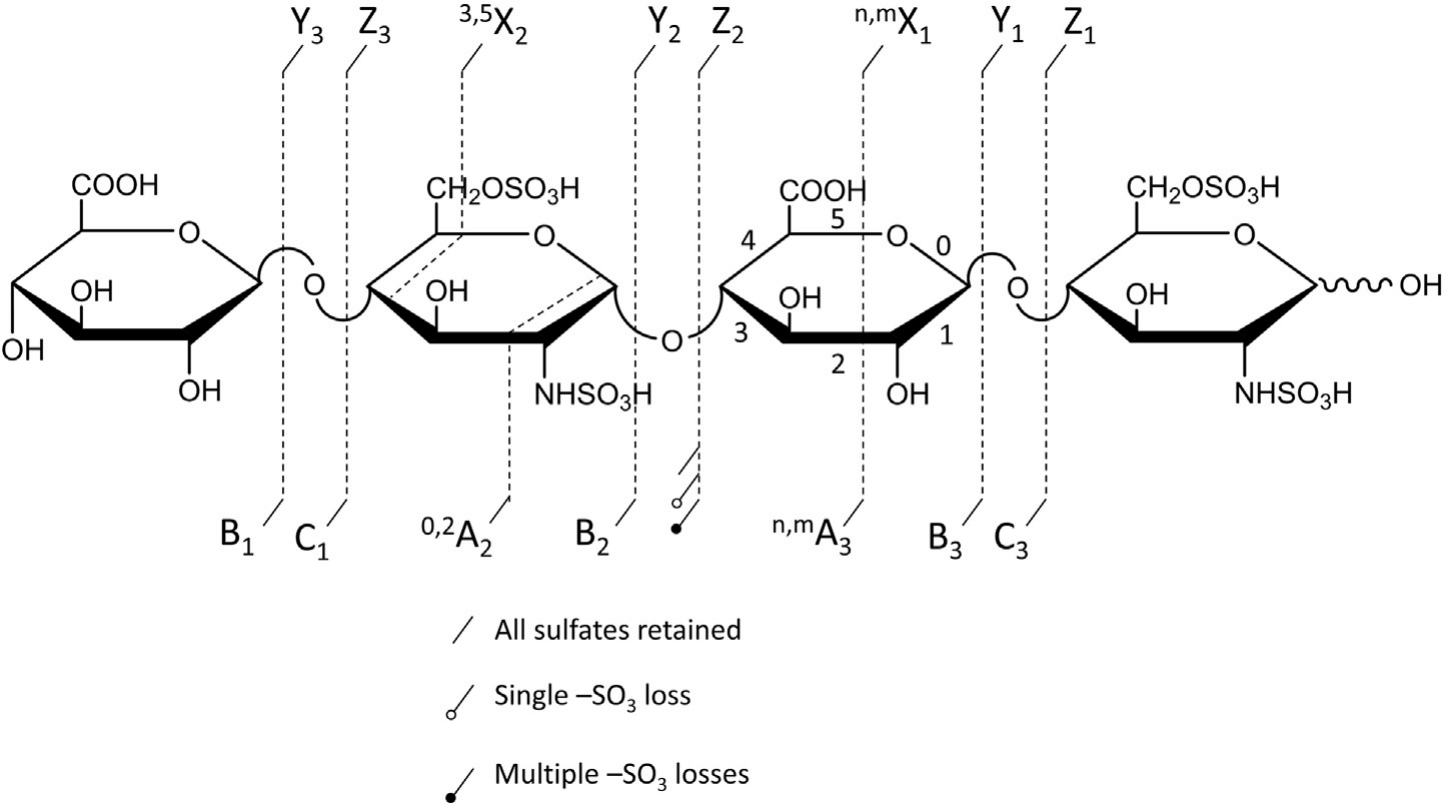
**Domon and Costello nomenclature for glycosaminoglycan fragmentation.** Key denotes symbols used for sulfate decomposition.

Tandem mass spectrometry (MS/MS) is a powerful tool for identifying the location of modifications within a GAG oligomer. Mass-selected precursor ions are activated and undergo fragmentation processes to yield a tandem mass spectrum. The fragment ions provide information that can be used to assign the structure of the precursor ion. The principal means of fragmenting a precursor ion are collisional activation, electron-based activation, ion–ion reactions, and photodissociation (74, 75, 76, 77, 78, 79, 80, 81, 82, 83, 84, 85, 86, 87, 88). The wide variety of available activation methods provide the means to fragment *via* many different reaction channels and can provide a range of structural details (Table 2). In general, there are two broad categories of fragmentation types, glycosidic bond cleavage and cross-ring cleavage, as shown in Figure 4. A series of ions from glycosidic cleavage between all residues provides composition information for each residue in a GAG chain, for example, the number of sulfo-modifications or the presence of *N*-acetyl in an amino sugar. Glycosidic cleavages give rise to fragments labeled B and C for fragment ions containing the nonreducing end of an oligomer or Y and Z for reducing end fragment ions (89). A pair of glycosidic product ions, *e.g.*, B and C, arise from fragmentation on either side of the oxygen atom that forms the glycosidic bond. The cleavage is accompanied with a hydrogen migration between the two fragments, ending on the oxygen of the cleaved glycosidic bond. For this reason, these complementary glycosidic cleavage products (B/C or Y/Z) differ in composition by H_2_O and in mass by 18 Da. This fixed mass difference facilitates the identification of such pairs of glycosidic cleavage products. One consequence of the hydrogen migration that accompanies glycosidic bond cleavage is the formation of a double bond on the residue that loses the hydrogen. Z-ions will have a double bond on their nonreducing end residue, and if this residue is a uronic acid, it will have the same composition as a Δ-uronic acid. For oligomers that have a Δ-uronic acid at their nonreducing end, it can be difficult to distinguish some Z-ions from C-ions because their composition and mass can be identical.

**Table 2:** List of activation methods commonly used for GAG characterization

MS/MS method	Pros	Cons
Collision-induced dissociation (CID)	• Easily accessible • Available on wide variety of spectrometers • Produces abundance of glycosidic cleavages	• Requires highly ionized precursor ion • Does not produce high abundance of cross-ring cleavages • High degree of -SO_3_ loss
Infrared multiphoton dissociation (IRMPD)	• Ample glycosidic cleavages	• Requires highly ionized precursor ion • Requires infrared laser • Multiple IR photons needed • Minimal cross-ring cleavages • Requires high-vacuum environment
Ultraviolet photodissociation (UVPD)	• Does not require highly ionized precursor • Single UV photon needed • High abundance of both glycosidic and cross-ring cleavages • Can be implemented on a variety of spectrometers	• Requires UV laser
Electron detachment dissociation (EDD)	• Does not require fully ionized precursor ion • High abundance of both glycosidic and cross-ring cleavages	• Long experiment time • Requires source of electrons • Typically implemented on FT-ICR MS • Requires multiply charged precursor ion
Electron-induced dissociation (EID)	• High abundance of cross-ring and glycosidic cleavages • Works on singly charged precursor ion	• Requires source of electrons • Requires singly charged precursor ion
Negative electron transfer dissociation (NETD)	• Can be implemented on a variety of spectrometers • Does not require fully ionized precursor ion • High abundance of cross-ring and glycosidic cleavages	• Shorter experiment times, can be implemented with separation techniques • Requires electron acceptor and carrier gas

The other category of fragmentation process, cross-ring cleavage, results from breaking two bonds within a monomer residue. The bonds are numbered as shown in Figure 4 and are denoted as ^n,m^A or ^n,m^X for nonreducing end and reducing end fragment ions, respectively (89). The superscripts n and m denote the specific bonds within a monomer ring that have been cleaved. Cross-ring fragment ions are useful for assigning the location of a modification within a sugar residue. The energy required to produce a cross-ring fragment is higher than that for glycosidic cleavage, because more bonds are broken in the former case. The choice of activation method influences the abundance of cross-ring *versus* glycosidic cleavage products and is an important consideration in selecting the method of analysis.

Collision-induced dissociation (CID) was one of the first fragmentation techniques to be applied to GAG sequencing (24, 87, 90). A collision with a neutral gas atom causes an increase in the internal energy of the precursor ion. The excess energy drives unimolecular dissociation by cleaving the most labile bonds, specifically the glycosidic linkages. In addition, sulfo-modifications undergo a facile rearrangement to release -SO_3_. CID leads to fragmentation by the lowest energy reaction pathway, which often is uninformative sulfate decomposition. The sulfate modification is most labile in its protonated form and is stabilized by deprotonation or metal cation–hydrogen exchange resulting in more informative cleavages (51, 88, 91, 92). However, adding a metal cation into the sample increases the complexity of the mass spectrum by introducing heterogeneity to the molecular ion region. Although cross-ring cleavages are not prevalent in CID spectra, highly ionized precursors can generate them as demonstrated for highly-sulfated HS/Hp oligomers by Kailemia *et al.* (91). In this work, sodium–hydrogen (Na–H) exchange was utilized to fully ionize the pentasaccharide Arixtra for sequencing studies.

CID is accessible on a wide variety of commercially available mass spectrometers. Guo *et al.* (93) used CID on an ion trap instrument in a multistep MS^n^ experiment to determine sequence information of highly sulfated GAGs. Chemical derivatization, specifically permethylation with stable isotope analogs, allowed the authors to determine site-specific sulfate location upon sequential MS/MS experiments performed in positive ion mode. Higher-energy collisional dissociation (HCD) is a similar type of collisional fragmentation found specifically on Thermo Fisher Scientific Orbitrap instruments. HCD, while still a low energy collision process differs from CID in that it occurs in a collision cell located after the C-trap in a Thermo Orbitrap instrument rather than within the linear ion trap as for conventional CID and allows observation of small product ions that fall below one-third of the *m/z* of the precursor, a limit of CID within the ion trap itself. CID and HCD fragmentations occur on the order of milliseconds making them suitable to combine with different separation techniques. Recently, Sharp *et al.* sequenced mixtures of chemically derivatized HS oligosaccharides using online LC and CID MS/MS (94, 95). Derivatization prevented loss of sulfate modifications and resulted in informative fragmentation upon collisional dissociation. Although CID does not produce a significant amount of cross-ring cleavages without additional modification in the form of metal cation–hydrogen exchange or derivatization, it can be a vital tool for analyzing modestly sulfated GAGs (1 or fewer sulfo-modifications per disaccharide) and combined with high-throughput separation experiments. Additionally, it has been shown that when analyzing oligosaccharides with Δ-unsaturated uronic acid at the nonreducing end, facile retro-Diels alder rearrangement occurs (96). This results in the formation of more cross-ring cleavages than when a saturated uronic acid is present at the nonreducing end (97).

**Fig. 5:**
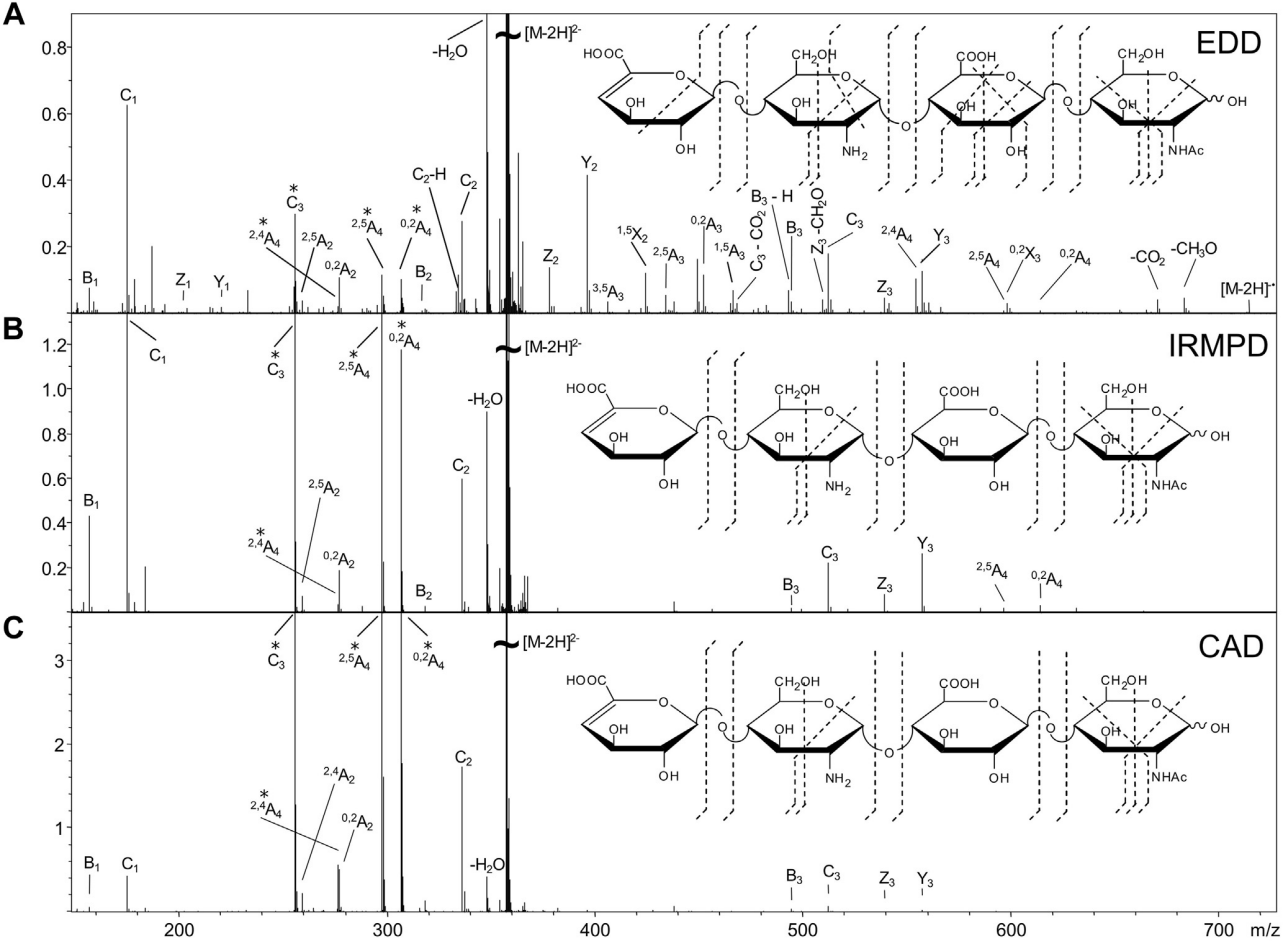
**Tandem MS spectra of [M-2H]**^**2−**^**of tetrasaccharide ΔUA-GlcN-GlcA-GlcNAc.***A*, EDD spectrum and cleavage map, *B*, IRMPD spectrum and cleavage map and *C*, CAD spectrum and cleavage map. Reprinted with permission from reference (84). Copyright 2007 American Chemical Society.

**Fig. 6:**
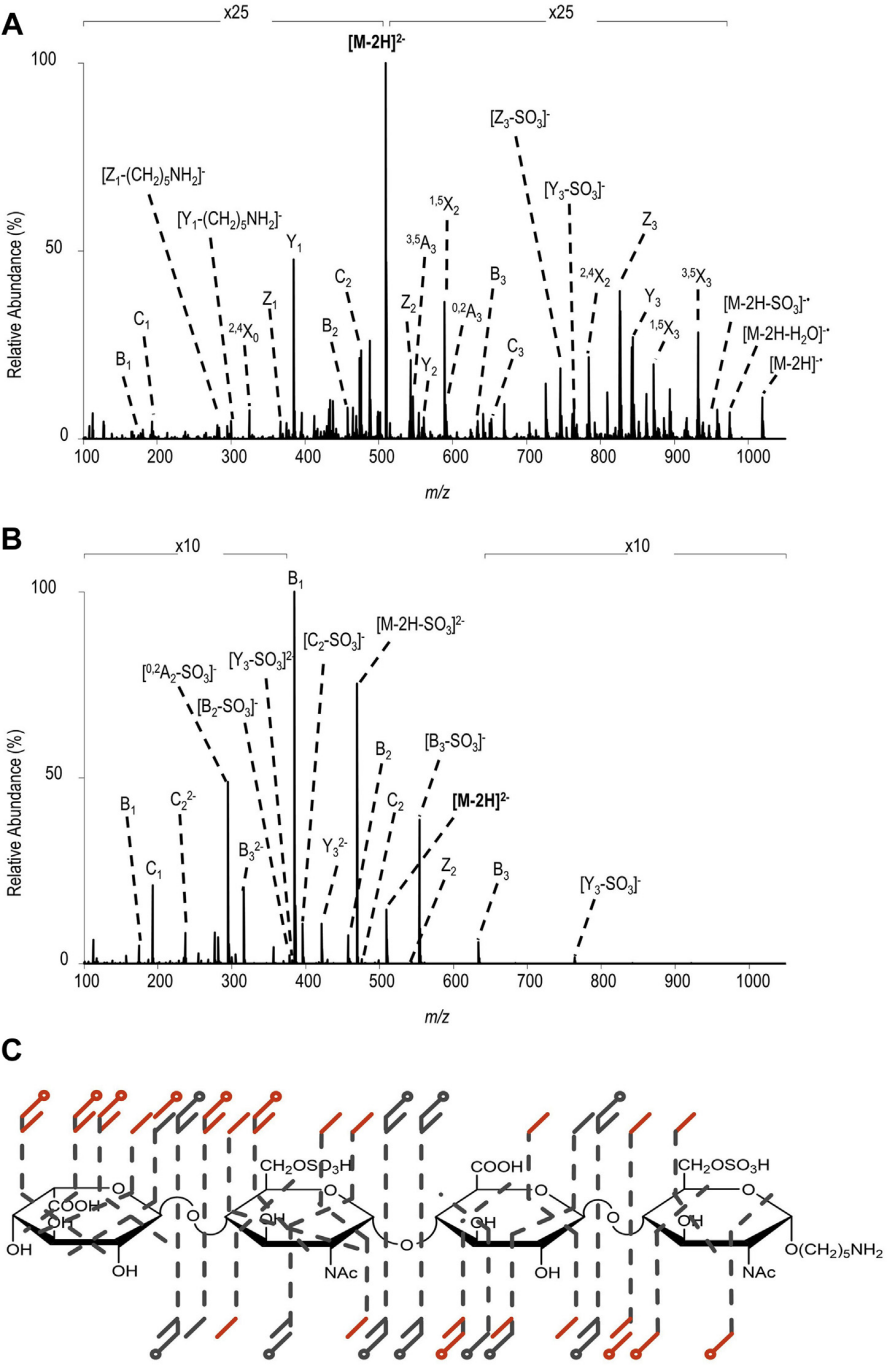
***A*, UVPD spectrum and *B*, HCD spectrum of [M-2H]**^**2−**^**(m/z 509) of disulfated tetrasaccharide IdoA-GlcNAc6S-GlcA-GlcNAc6S-(CH**_**2**_**)**_**5**_**NH**_**2**_**.***C*, annotated structure showing fragment ions for both UVPD and HCD, with fragments only seen in UVPD outlined in red. Reprinted with permission from reference (59). Copyright 2019 American Chemical Society.

Photodissociation is another ion activation approach for assigning the structure of GAGs. Infrared multiphoton dissociation (IRMPD) produces ample glycosidic cleavages in GAGs as well as other types of glycans (52, 84, 98, 99). IRMPD typically uses an infrared laser such as the 10.6 μm output of a CO_2_ laser, to raise the internal energy of trapped ions through the serial absorption of infrared photons (100). Absorption of a single IR photon merely raises the vibrational energy of the precursor. In order to access a dissociative excited state, many IR photons must be absorbed, as implied by the name infrared *multiphoton* dissociation. Activation by IRMPD is a threshold process that accesses the lowest energy fragmentation pathways. As with CID, IRMPD yields minimal cross-ring cleavages and a high degree of –SO_3_ loss for protonated sulfate modifications. Wolff *et al.* (75) showed that IRMPD produces similar fragmentation to CID in GAG standards, illustrated in Figure 5, *B* and *C*. IRMPD requires a fully ionized precursor to produce informative fragmentation. This can be achieved by choosing a high charge precursor or by exchanging a metal cation such as Na^+^ for protons in ionizable functional groups (sulfate and carboxylate). McClellan *et al.* (101) demonstrated the importance of precursor selection when using IRMPD. Different charge state precursors were shown to produce different fragment ions, with higher charge states being preferred. This phenomenon has been shown when using collisional methods as well. IRMPD is most frequently implemented on a Fourier Transform ion cyclotron resonance mass spectrometer (FT-ICR MS), as it requires a high-vacuum environment during the multiple steps of photon absorption, to avoid collisional relaxation of the intermediate photoexcited states. More recently, ultraviolet photodissociation (UVPD) has been used for the analysis of GAG standards (74). UVPD uses an ultraviolet laser to raise the internal energy of trapped ions, resulting in fragmentation (102). Unlike IRMPD, a single UV photon is adequate to raise the precursor ion into a dissociative state. Racaud *et al.* (103) used UVPD in the 220 to 290 nm range to dissociate heparin-derived disaccharide dianions. This favored informative cross-ring fragments and yielded electron-photodetachment ions as well as the corresponding charge-reduced neutral loss products. This study also demonstrated the importance of deprotonated sulfo-modifications for informative fragmentation (103). Klein *et al.* (74) showed that UVPD at either 193 nm or 213 nm produced both glycosidic and cross-ring fragmentation in GAG standards ionized in negative mode, while maintaining sulfate modifications. As demonstrated by Klein *et al.*, (74) UVPD does not require a fully ionized precursor to produce informative fragmentation. An HS tetramer with deprotonation of only two of its four ionizable sites yields cross-ring and glycosidic cleavage with minimal sulfo-decomposition, as shown in Figure 6. UVPD works well with ion trap instruments as there is no requirement for high vacuum, in contrast to IRMPD (102).

Electron-based activation methods play a significant role in contemporary biological mass spectrometry. Electron detachment dissociation (EDD) has been widely used for the analysis of GAG chains (52, 74, 75, 80, 81, 82, 84, 85, 86, 98, 104). EDD operates by irradiating multiply charged negative ions with 15 to 20 eV electrons. This causes ion activation *via* electronic excitation and promotes electron detachment and radical formation, with the production of both even- and odd-electron fragment ions (75). Unlike vibrational-activation methods such as CID and IRMPD, EDD yields a large quantity of cross-ring cleavages. In the past, EDD was restricted primarily to FT-ICR MS due to the need to trap ions in a static electric field during electron bombardment. EDD of GAGs was first applied to HS tetrasaccharide standards with a modest degree of sulfation and was found to produce far more fragmentation products than IRMPD or CID, as shown in Figure 5 (75). This technique has since been expanded and used for longer, more highly sulfated GAGs (28, 82, 84, 85, 86, 98). Wolff *et al.* (77) showed the capability of EDD to distinguish epimeric HS tetrasaccharides that differ only by C-5 stereochemistry in the uronic acid closest to the reducing end. Agyekum *et al.* (82) developed a diagnostic ratio for assigning C-5 stereochemistry in a diverse pool of HS tetramers. Leach *et al.* (80) investigated the importance of precursor selection using synthetic Hp/HS tetramers with 1 to 2 sulfates per disaccharide unit. EDD produced the best results when the degree of ionization equaled one more than the number of sulfate modifications. The addition of sodium counter ions was used to create ionized carboxyl groups to increase the likelihood of electron detachment from the carboxylate for highly sulfated GAGs (80). Electron-induced dissociation (EID), which irradiates singly charged anions with 6 to 20 eV electrons, activates ions by electronic excitation (79). EID produces similar fragmentation to EDD, but without going through the process of electron detachment. Wolff *et al.* (79) showed that EID produces an abundance of cross-ring fragmentation, resulting in EDD-like fragmentation. The presence of cross-ring fragmentation primarily within hexuronic acid residues in both EID and EDD suggests that these residues are more labile when activated *via* electronic excitation (79).

**Fig. 7:**
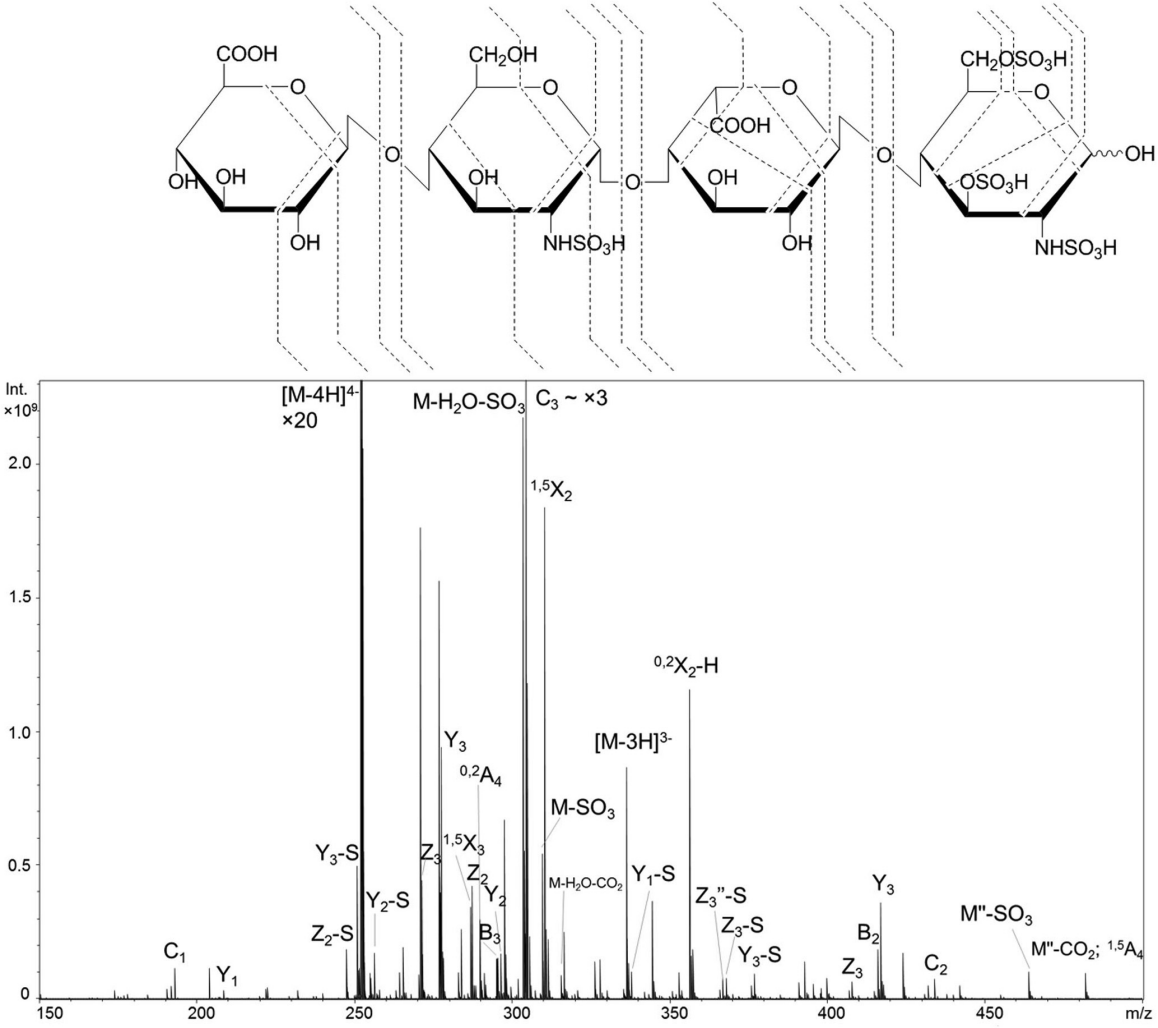
**NETD spectrum of [M-4H]**^**4−**^**of tetrasulfated tetrasaccharide GlcA-GlcNS-IdoA-GlcNS3S6S and cleavage map illustrating the ability for NETD to distinguish 3-O sulfation from 6-O sulfation with the presence of**^**0,3**^**A**_**4**_**.** Reprinted with permission with reference (96). Copyright 2018 American Chemical Society.

Negative electron transfer dissociation (NETD) is another useful ion activation technique for GAGs (105, 106). NETD is the negative complement to electron transfer dissociation (ETD). This ion–ion reaction involves gas-phase electron transfer from a multiply charged anion precursor to a reagent cation (107). Commonly, this reagent species is fluoranthene radical cation; however, Xe^+^ or SF_5_^+^ can also be used (106, 108). Like EDD, NETD produces a radical intermediate that fragments to both even- and odd-electron products. NETD of GAGs was originally demonstrated with ion trap MS but can also be implemented with FT-ICR MS and Orbitrap MS (105, 106, 109, 110). The electron transfer process in NETD occurs rapidly, on the order of 10 to 100 ms. This compares quite favorably to EDD, which generally uses activation times of 1 s. The short reaction time for NETD allows it to be paired with online separation techniques such as high-performance liquid chromatography (HPLC) and capillary zone electrophoresis (CZE). Leach *et al.* (110) showed the ability to produce informative fragmentation of GAGs on a 10 ms timescale using NETD. Wu *et al.* (111) used NETD to distinguish 3-*O versus* 6-*O* sulfation in the amino sugar residues of HS oligomers up to dp6 in length. Figure 7 illustrates the capability of NETD to produce cross-ring fragment ions that identify the location of all sulfation modifications in an HS tetramer, with a precursor that is deprotonated at only four of its six ionizable sites. The ability to distinguish 3-*O* from 6-*O* sulfation with a less than fully ionized precursor is promising for incorporation with online separations, where one has less control over the charge state of the precursor ion than with infusion of a sample.

## Separations of Complex Mixtures

GAGs that are extracted from biological samples are inherently complex and heterogeneous due to their nontemplate biosynthetic pathway (2). Typically, full-length glycans are processed by enzymatic or chemical partial depolymerization and processed with preparative scale separation methods to isolate oligomers of a certain length. Such samples are still complex mixtures and may be further resolved by ion exchange chromatography to separate components by their degree of sulfation. The resulting fractions are mixtures of isomeric GAGs differing only in the location of modifications. These are challenging to analyze using direct infusion, as isomers cannot be resolved by mass-to-charge filtering. Online separation methods are essential for resolving complex mixtures into their various components. Some approaches for separating GAGs coupled to MS include HPLC, hydrophilic interaction liquid chromatography (HILIC), ion mobility spectrometry (IMS), and CZE (Table 3) (73, 112, 113, 114, 115).

**Table 3:** Separation methods commonly used to isolate GAG mixtures

Separation method	Pros	Cons
Size-exclusion chromatography (SEC)	• Separate chain lengths by increments of dp2 • Simple and robust	• Multiple species with different degree of sulfation can elute at same time • Ion suppression is often needed
Strong anion exchange (SAX)	• Separates based on charge • Can isolate different degree of sulfation	• High number of inorganic salts resulting in contamination
Reverse-phase ion pairing (RPIP)	• Can separate similar GAGs	• Ion pairing reagent is needed • Unable to separate highly polar and ionic compounds
Hydrophilic interaction liquid chromatography (HILIC)	• Polar stationary phase • Separates based on polarity	• Longer equilibration time than reversed-phase LC • Mobile-phase pH shift can affect retention times
Ion mobility spectrometry (IMS)	• Fast separation • Occurs after ionization • Can be used with direct infusion	• Instrument specific
High field asymmetric waveform ion mobility spectrometry (FAIMS)	• Separates spatially and by differential mobility • Easily coupled with slow MS acquisition	• More time needed for ion accumulation
Capillary zone electrophoresis (CZE)	• Separates based on size, shape, and charge • Can distinguish isomers, including C-5 stereochemistry	• Sensitive to salt • Requires interface for pairing with MS

**Fig. 8:**
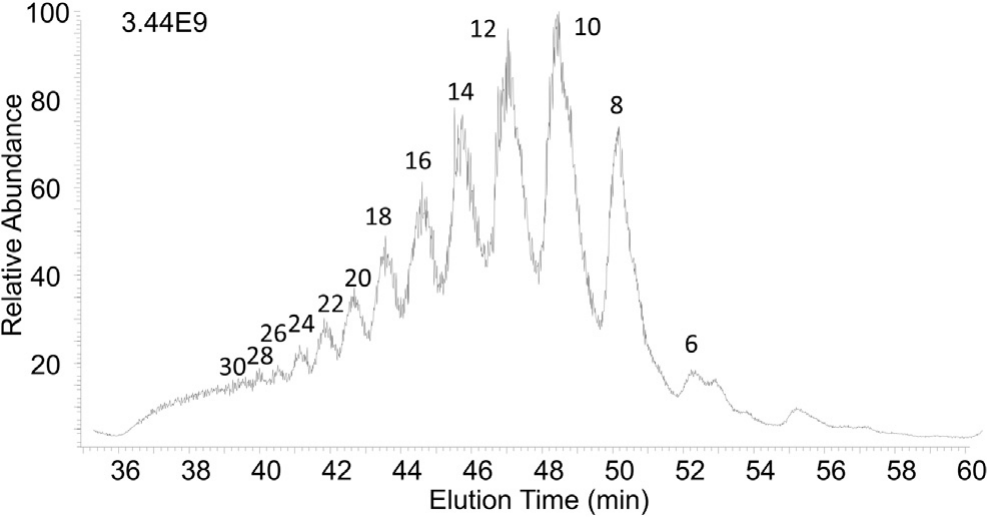
**SEC-IS-MS total ion chromatogram of dalteparin.** Size of oligomer is indicated for each chromatographic peak. Reprinted with permission from reference (101). Copyright 2016 American Chemical Society.

HPLC covers a broad range of separation techniques including size-exclusion chromatography (SEC), strong anion exchange (SAX), reverse-phase ion pairing (RPIP), and HILIC. These techniques are ideal for separating oligosaccharides with different degrees of polymerization. However, in most cases they fall short for separation of GAG isomers without additional sample preparation. SEC is commonly used as the first purification step to separate oligosaccharides into various chain lengths by increments of dp2. It is a simple and robust separation technique that provides profile information about compositions within the mixture, but it often presents multiple species with various sulfation patterns eluted at the same time. Zaia *et al.* and Zhang *et al.* performed experiments to separate complex mixtures of LMWHs into chain lengths ranging from dp2 to dp30 (116, 117). Figure 8 depicts the separation of the LMWH dalteparin using SEC combined with ion suppression to elute GAG chains of different sizes for MS analysis (116).

SAX provides another step of purification for GAGs by separating molecules based on their charge. Thus, GAGs with different numbers of sulfate modifications can be isolated. However, SAX can be challenging to perform with online mass spectrometry due to the high amount of inorganic salts used for separation. MS contamination is common with this technique, but several groups have worked to reduce the type and abundance of salt present after separation (111, 116, 118). Miller *et al.* (119) combined SEC and SAX to separate GAG oligosaccharides into fractions using volatile ammonium bicarbonate to reduce contamination.

RPIP is another form of LC used to separate mixtures of GAGs prior to MS analysis. An ion pairing reagent is added to the mobile phase and interacts closely with the sulfate groups on the GAG, rendering them neutral in charge so that they are retained on a reverse-phase column. Typically, ion pairing reagents are organic compounds, such as di- and tributyl amines (120). Although this separates similar GAG species, it can reduce ionization yield and complicate the MS and MS/MS analyses (93, 120, 121, 122). RPIP is suitable for the off-line separation of GAGs prior to analysis, but is not well suited for online analysis.

HILIC has been used for online separation of GAGs with subsequent mass analysis. It is different from reverse-phase HPLC in that it uses a polar stationary phase, which can retain polar or ionized analytes. Molecules are separated based on their polarity, a useful attribute for the analysis of highly anionic GAGs. The mobile phase typically used for HILIC separations of GAGs is acetonitrile and water, which is well matched for ESI-MS. HILIC does not require ion pairing reagents, simplifying sample preparation and increasing MS sensitivity. Several groups have analyzed GAG oligomers up to dp30 using HILIC-LC-MS (65, 123, 124). Using a maltose-modified HILIC column and high-resolution MS, Sun *et al.* (125) separated and identified 36 building blocks that comprise the nitrous acid depolymerized LMWH mixtures, dalteparin and nadroparin. Over 30 building blocks were separated within 1 h without derivatization of the oligosaccharides. A combined method of HILIC LC-NETD MS/MS was recently reported in which chemically synthesized tetra- and hexasaccharide isomers were separated and sequenced without permethylation (118). To improve precursor sensitivity, an ion suppressor was implemented prior to MS analysis to reduce the abundance of salt present after separation. The GAG species were then fragmented with NETD and produced glycosidic and cross-ring cleavages that could be used to assign their structures.

**Fig. 9:**
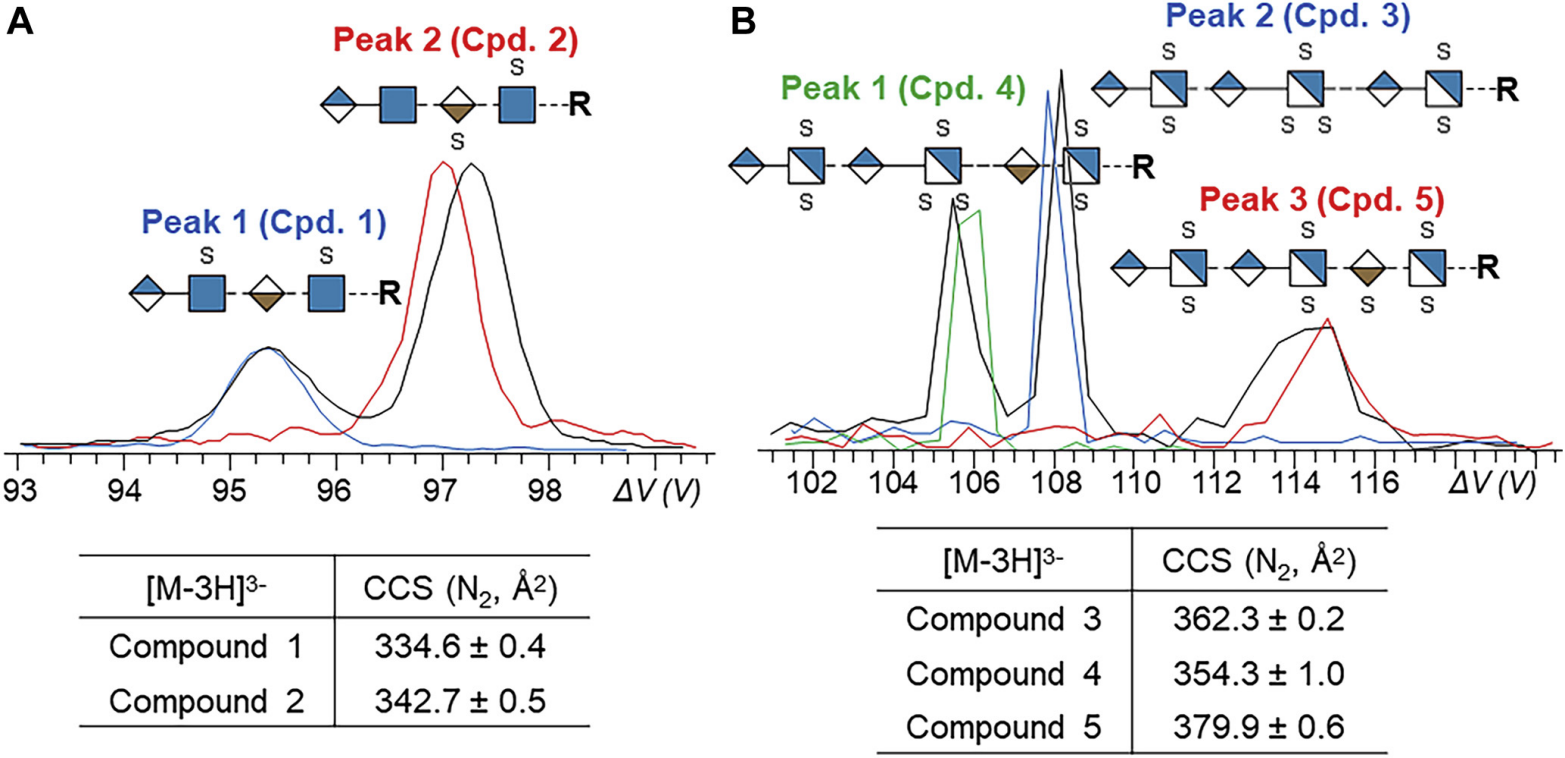
**Gated-TIMS separation of dp4 and dp6 isomers.***A*, extracted ion mobility spectra ([M-3H]^3−^) of Compound 1 (*blue trace*), Compound 2 (*red trace*), and their mixture (*black trace*). Extracted ion mobility spectra ([M-3H]^3−^) of Compound 3 (*blue trace*), Compound 4 (*green trace*), Compound 5 (*red trace*), and their mixture (*black trace*). Reprinted with permission from reference (110). Copyright 2019 American Chemical Society.

With heterogeneous GAG mixtures, all types of compositions may be present, including ones with different numbers of sulfo-modifications as well as ones with the same number, such as isomers and diastereomers. While HILIC can separate molecules of different composition, it does not adequately separate isomeric GAGs. Other methods have been implemented to address this issue. Specifically, IMS and CZE can separate isomeric GAGs and even diastereomers. Gas-phase separation using ion mobility is fast and occurs after ionization. IMS separations occur on the order of milliseconds, whereas LC and CZE separations can take minutes or hours. IMS separation can be used in tandem with direct infusion using conditions to reduce adduct formation, which reduces the complexity of analysis. Wei *et al.* (126) recently separated and analyzed highly sulfated GAG isomers using gated-trapped IMS (gated-TIMS) combined with NETD. Using gated-TIMS, stereoisomers were separated, as shown in Figure 9, and diagnostic ions produced from NETD confirmed their structure. Daniel and coworkers have combined traveling wave ion mobility spectrometry (TWIMS) in combination with tandem mass spectrometry to separate and characterize mixtures of CS oligomers (127).

An alternative ion mobility approach to separating isomeric GAG oligomers is differential mobility spectrometry (DMS), also known as high field asymmetric waveform ion mobility spectrometry (FAIMS). FAIMS separates ions spatially and is a scanning method that filters ions by their differential mobility. It couples well with slow MS acquisition methods such as FT-ICR MS. Amster and coworkers used FAIMS to separate isobaric mixtures of oligosaccharides followed by structural characterization using EDD (104). Another interesting area of development is a combination of IMS-MS with cryogenic IR spectroscopy. The Rizzo group has demonstrated separation of isomeric CS and HS GAG disaccharides using this technique (128). Some of the isomers have similar drift times, but with unique fingerprint IR spectra, it is possible to distinguish the different types of disaccharides. Overall, there are multiple types of IMS that can be utilized to distinguish isomeric oligosaccharides on the millisecond timescale for high-throughput applications.

**Fig. 10:**
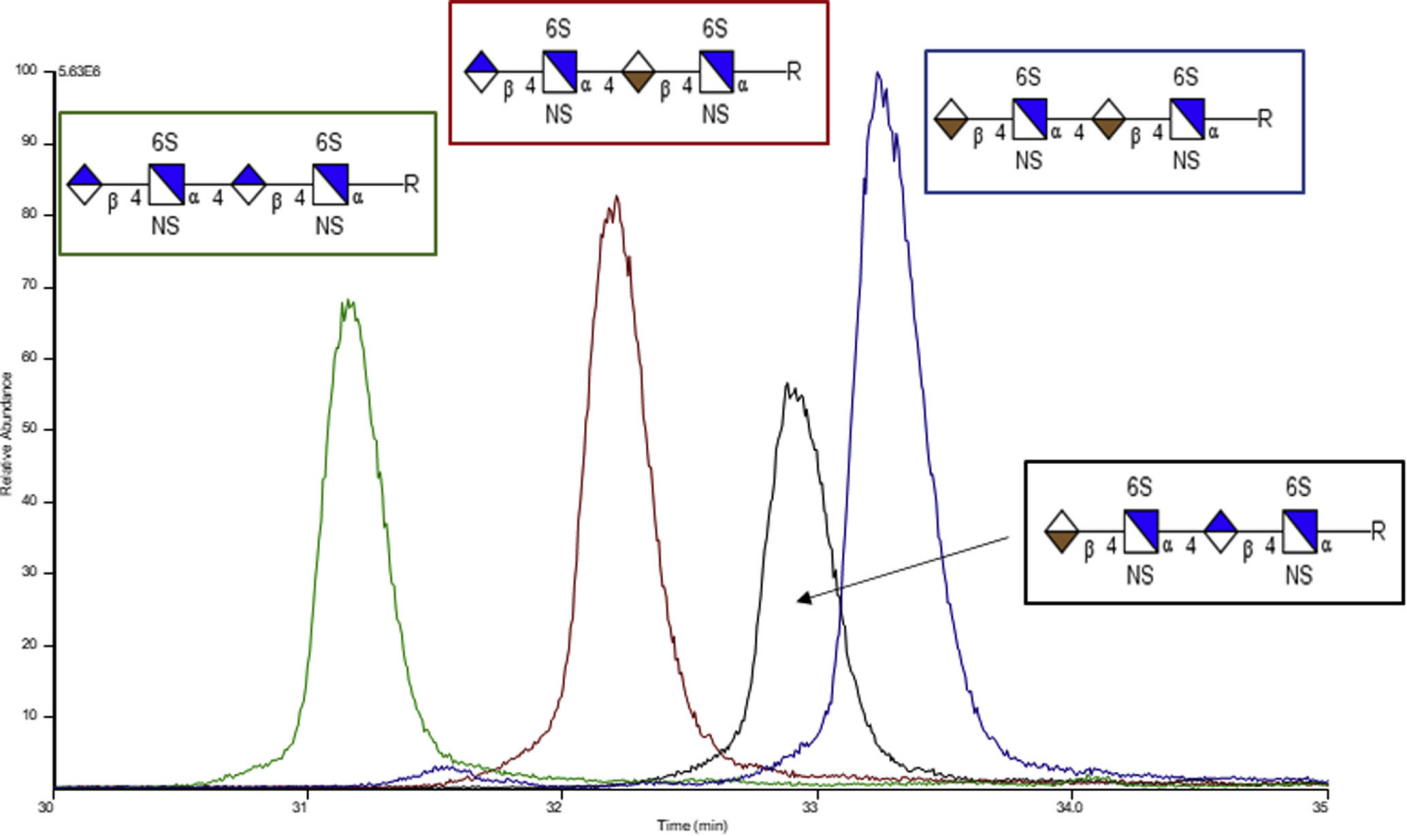
**Capillary zone electrophoresis (CZE) separation of dp4 epimers**.

CZE is well suited for sulfated GAGs due to their ionic nature, as it separates components based on their charge, size, and shape. The majority of initial work completed on GAGs was performed in normal polarity mode, which resulted in longer migration times (83, 129, 130, 131). Recent studies were performed using reverse polarity, in which a negative potential is applied to the separation capillary, to facilitate faster separation and improve resolution of CZE separated GAGs. Initial work focused on disaccharides and later progressed to oligosaccharides. CZE-MS analysis has been used to assign the degree of polymerization, sulfo-modification, and to show the presence of isomers (71, 132). An example of this is shown in Figure 10; four tetrasaccharide epimers were separated based on difference of the C-5 stereochemistry on the uronic acid residues. Recently NETD was used to characterize CZE separated HS tetrasaccharide standards and the LMWH pharmaceutical enoxaparin (109). Ion activation methods compatible with the CZE timescale, such as CID/HCD and NETD, have been incorporated into CZE separation experiments (109).

Each of these separation techniques was developed to simplify the analysis of GAG mixtures. With these techniques combined with MS and MS/MS, researchers are better equipped to investigate and solve applied biological and pharmaceutical problems. Nevertheless, further development is necessary to improve the speed, sensitivity, and capability of distinguishing the components within a heterogeneous GAG sample.

## Glycosaminoglycan Software Developments

GlycoWorkbench is a widely used software tool for the analysis of carbohydrate mass spectra and tandem mass spectra (133). GlycoWorkbench provides a GUI that allows one to construct glycan structures using the building blocks of all known glycans and modifications. The user can build any type of glycan molecule desired and calculate the masses of all possible fragments for comparison with experimental data. In the absence of software, assigning fragment ions to features in a carhohydrate structure is tedious and time-consuming. GlycoWorkbench has greatly benefited the assignment process for MS/MS spectra of GAG standards, for which the structures are already known. When working with unknown samples, one must consider all possible isomers; as the sample molecule becomes larger, the possibilities become exponentially greater. The large number of possible compositions, structures, and fragments that must be considered for assignment of an unknown structure have led researchers to develop software to automate the analysis of GAG tandem mass spectra.

Assignment of GAG composition is typically the first step in MS analysis. Software designed to rapidly assign glycan compositions from accurate mass measurements have been developed and are particularly useful for experiments with large MS data sets such as those produced by online separations of mixtures. Manatee was designed by the Zaia group to rapidly extract, assign, and compare glycan compositions from complex LC-MS datasets (134). The Zaia group then developed GlycReSoft, which added noise reduction and confidence measurements to the efficient data analysis of Manatee (135). Recently developed programs GlycCompSoft and GRITS Toolbox have made composition assignment software more user-friendly with features that automatically assign peaks, allow postprocessing, and facilitate more specific experimental analysis (136, 137).

The next step after composition is automated characterization of GAG structures from MS/MS data. Ion activation allows researchers to pinpoint modifications to specific residues or sites within a residue. Determining specific sites of modification within GAG molecules is essential to understanding GAG–protein binding and biological function. The simplest way to assign structural characteristics is through brute force methods that generate all possible fragments and match theoretical fragments with experimental data (138, 139, 140). Even with the processing power of modern computers and CPUs, this method can take an exceedingly long time for larger GAG precursors. More sophisticated software has begun to rely on expertly crafted algorithms to determine GAG structures quickly and confidently. GAGfinder, created by Hogan *et al.*, is a brute force algorithm that compares experimental MS/MS data with a list of theoretical sequences and fragments generated from the precursor to determine GAG structures as well as composition assignment. GAG-ID was developed by Chiu *et al.* to automate assignment of derivatized Hp/HS that are separated and fragmented with LC-MS/MS (138, 139). Duan *et al.* utilized a genetic algorithm that scores fragment and sequence fitness without an exhaustive database search to significantly reduce processing time (141, 142). Many groups throughout the world are working to create and unify databases, such as GlyTouCan, for glycan structures and MS/MS data to facilitate widespread analysis (143). These advances in analysis software are making it easier for researchers to tackle real-world problems involving GAGs.

## Applications

Mass spectrometry has been utilized for decades to tackle a variety of biological targets, including GAGs. Initially, most of the work focused on using a bottom-up approach in which enzymatic digestion of the GAG is performed prior to MS analysis to reduce the complexity of the sugars (90, 144, 145). Disaccharide analysis is still performed routinely to statistically determine the components and disaccharide backbone motifs of longer chains, but it results in a loss of structural information such as linkage, order, and sulfation patterns (145, 146). However, the location and organization of modification patterns on GAGs dictate their biological activity. Thus, the most recent endeavors have focused on partially digested sugars that retain biological function and even full-length glycan chains.

LMWHs are partially depolymerized heparin and are important pharmaceutical compounds. In 2008, there were a number of complications associated with contaminated heparin (147). Since then, there have been a multitude of experiments focused on analyzing the composition of pharmaceutical heparins (148). Enoxaparin, dalteparin, and other versions of the LWMH drugs are produced through different depolymerization procedures. These heterogeneous mixtures range from dp2 to dp30 with large variation in sulfation and sequence composition (125, 149, 150). As one of the most sensitive analytical techniques, MS is well suited for this type of analysis, particularly when HPLC or CZE is used to separate the mixtures (131, 144). The longer chains in LMWH pharmaceuticals have been analyzed using LC-MS by Linhardt and coworkers to determine the major structures present (123, 151, 152). Over 80 compositions have been detected with these methods. Studies using CZE-MS have also found similar results (71).

CZE paired with NETD MS/MS has recently been used to determine the composition of GAGs found in human urine (153). This work looked at urine from both males and females, separated into two age groups (young adults aged 23–25 and adults aged 35–45). These groups only represent a small number of nondiverse individuals and were not controlled for diet, hydration level, or evaluated for health. It was found that female urine for both age groups had higher levels of HS than males (75.7% and 68.1%, respectively), and males had higher levels of CS than females (31% and 24%, respectively) (153). For both males and females, it was found that young adults had a higher level of HS, whereas older adults had higher levels of CS. Disaccharide analysis based on LC-MS multiple reaction monitoring (MRM) identified unsulfated (0S) as the predominant HS disaccharide and 4-*O* sulfated (4S) as the predominant CS disaccharide (145, 153). Though these were the most predominate sulfation patterns found, a wide range of sulfation patterns for both HS and CS was seen. Further analysis by molecular weight analysis and CZE-MS/MS found oligosaccharides ranging from dp2 to dp20 (153).

Of particular interest to biologists is the specificity of the interaction of a GAG with its target protein and how this specificity relates to the specific structural features of a GAG chain. MS has played a significant role in probing the interactions of GAG with their protein targets. MS has the advantages of low sample consumption and a tolerance for GAG heterogeneity compared with other analytical methods for such studies, for example, NMR spectroscopy. Kaltashov and coworkers have studied the binding stoichiometry of unfractionated heparin with antithrombin III (ATIII) by using native-spray mass spectrometry (154). Native spray uses electrospray ionization of nondenaturing solutions of proteins and ligands to produce gas-phase complexes that are indicative of solution-phase behavior. These researchers were able to derive information such as glycan chain length and protein–glycan stoichiometry using an unfractionated heparin sample. This gas-phase approach was extended to studies of the ATIII/Factor Xa complex, which is stabilized by its interaction with heparin (155). Another application of native-spray mass spectrometry is to examine changes in higher-order protein structure upon complexation with a GAG ligand. Such studies are facilitated by the application of ion mobility mass spectrometry (IMS-MS). The known conformational change that occurs in solution for ATIII upon binding the pentasaccharide Arixtra was found to be preserved in the gas phase by using IMS-MS (21). These studies confirmed that specific binding to ATIII requires certain elements of sulfo-modification, and by removing some of these, both the binding efficiency and specificity were found to decrease. This approach has been extended to studies of other GAG binding proteins, including the fibroblast growth factor and the roundabout protein (156, 157).

Expanding upon past work, which digested HS from bovine brain tissues, Zaia and coworkers demonstrated the ability to detect and analyze GAGs, N-glycans, and proteins from histological tissues (158, 159). By profiling different glycan classes as well as proteins, more detailed information can be obtained regarding temporally and spatially regulated tissue phenotypes (159). A workflow was developed in which fixed or fresh tissue can be digested to yield GAGs, N-glycans, and proteins at once. This involved sequential enzymatic digestion by hyaluronidase, chondroitinase ABC, heparin lysases, trypsin, and PNGase F to the same area of interest (159). Products were then analyzed using LC-MS. For GAGs, it was found that digestion time can be reduced by more than half (200 min–50 min) when using microwave-assisted tissue digestion compared with incubator digestion. When investigating fresh and fixed mouse brain and liver samples, HA, CS, and HS were in the mouse brain samples, whereas only HS was in the liver samples. It is known that HA and CS liver expression in rats is only 5 to 10% of that expressed in brains, which could explain why only HS was in the liver (160). It was also determined that a tissue spot size as small as 0.5 μl (1 mm) could be used for GAG digestion (159). Therefore, a specific area of tissue can be analyzed as opposed to bulk tissue analysis, allowing for analysis of both pathological and nonpathological sample regions (159).

Recent studies of a CS binding protein have displayed a potential for this recombinant protein to facilitate the delivery of anticancer compounds into the tumor environment (161). The malarial protein VAR2CSA binds to distinct types of CS that were until recently thought to be exclusively found in the placenta. However, this same CS is found in malignant cells and can be targeted by recombinant VAR2CSA (rVAR2) (161). To determine the structure of the CS found in both placenta and malignant tumors, disaccharide analysis was done using chondroitinase ABC and SEC-MS (162). Collisional energy was applied for MS/MS to determine sulfation position of disaccharides (163). SEC-MS results showed that for bovine trachea CS, 90% of the compounds identified were mono-sulfated and 10% were unsulfated. In contrast, cancer-associated CS was 98% mono-sulfated (161). MS/MS results showed that of the 90% mono-sulfated CS, 79.6% was 4-*O* sulfated and 20.4% was 6-*O* sulfated (for lymphoma cells, 4-*O* and 6-*O* sulfation was found to be 69.8% and 30.2%, respectively) (161). Further studies determined that 17 proteins, including syndecan 1, carbonic anhydrase IX, CD44, and CS-A modified proteoglycan CSPG4, can carry placental CS when overexpressed. Primary human tumor specimens representing 17 major human cancer types were tested to determine the intertumor diversity in expression of PGs able to display placenta CS. This placental CS was differentially, yet complementarily expressed in each of the 17 cancer groups tested (161). The interaction of rVAR2 with the CS-modified form of CD44 in melanoma cells was validated. rVAR2 pulled down glycosylated CD44 from melanoma protein lysates. These data suggest that rVAR2 can be used to broadly target placental CS chains in human malignancies with differing PG expressions (161). Further studies on the ability for rVAR2 to target tumor cells are ongoing (164, 165).

**Fig. 11:**
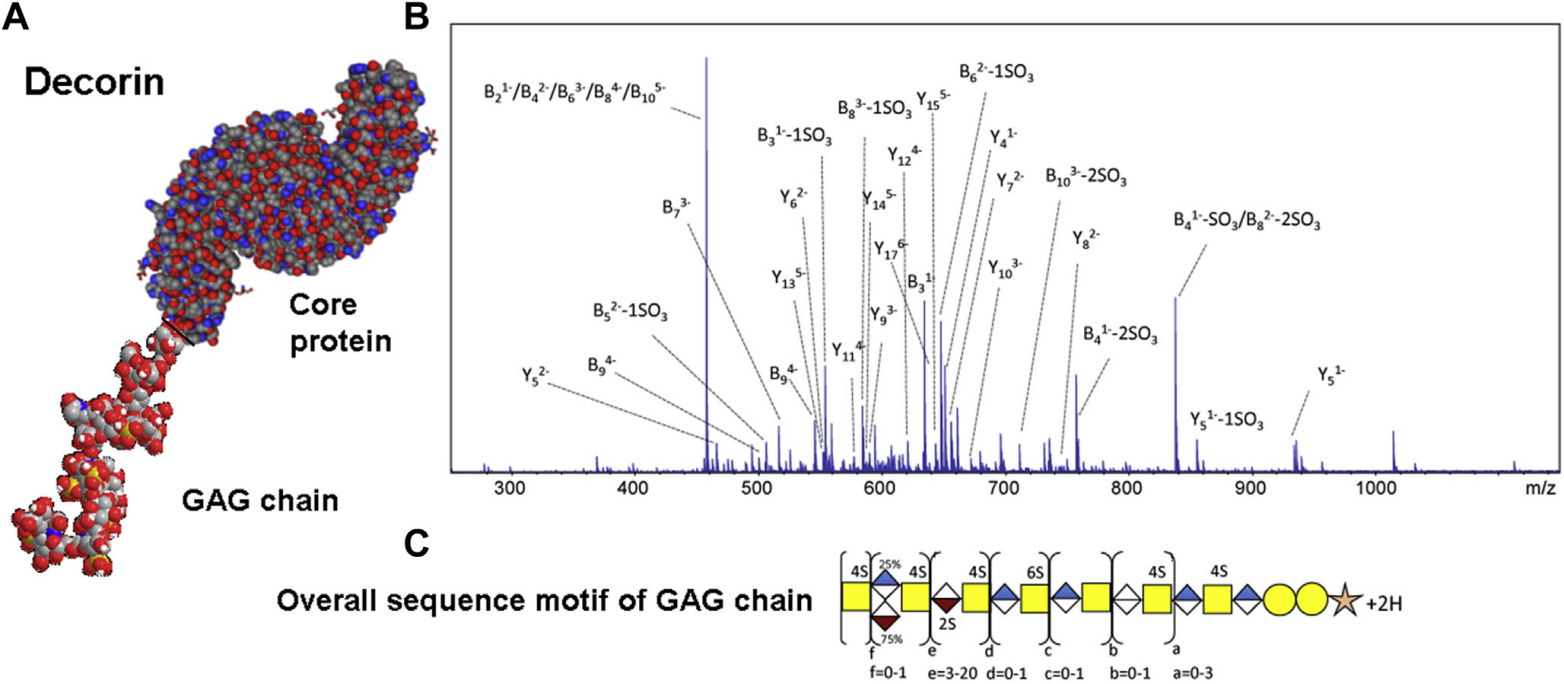
**Modeled structure and motif of decorin glycosaminoglycan.***A*, space-filled structure of decorin PG, with the core protein from PDB (1XCD). Carbons (*gray*), hydrogens (*white*), oxygens (*red*), nitrogens (*blue*), and sulfurs (*yellow*) are shown. The O-linked GAG chain (dp20–8S) is shown with the reducing end (RE) and nonreducing end (NRE). *B*, CID tandem mass spectrum of decorin GAG chain dp20 with 7 sulfo-modifications. *C*, structural motif for decorin GAG chains determined by MS. Reprinted with permission from reference (23). Copyright 2017 American Chemical Society.

As an alternative to the bottom-up approaches to GAG characterization described above, there is a small body of work on the top-down analysis of intact glycan chains isolated from PGs. The simplest PGs, bikunin and decorin, have been the subject of this approach, with the GAG chains (CS) being analyzed using high-resolution mass spectrometry (23, 30, 31, 32). Though it is easier to analyze digested GAG chains, intact decorin and bikunin GAG chains have been analyzed (30, 31, 32). For bikunin, the PG fraction was proteolyzed by actinase E digestion to yield a serine terminated glycan that was isolated by strong anion exchange spin columns (30, 31, 32). For decorin, the GAG component was released by base-catalyzed β-elimination under reducing conditions (30). The resulting heterogeneous mixture of GAGs released was separated into fractions of different chain lengths by a series of steps including size-exclusion chromatography, strong anion exchange, and polyacrylamide gel electrophoresis (PAGE) (30, 31, 32). Chains up to dp45 have been purified and analyzed using these techniques. Both bikunin and decorin have a single CS/DS GAG chain attached to a core protein; bikunin has a CS chain, whereas decorin has a longer DS chain. The Linhardt and Amster groups collaborated for these challenging studies, using several stages of purification to fractionate the full-length glycans prior to MS analysis, as well as high-resolution MS to examine the unfractionated mixture of intact glycans. The fractions were then analyzed by both Orbitrap MS and FT-ICR MS instruments using MS for composition and CID/HCD MS/MS for sequencing. These analyses found the complexity of these mixtures to be far lower than anticipated for a random distribution of modifications (23, 30, 32). For bikunin, a conserved pattern of modification was observed for all the glycans that were analyzed (dp27-dp43). Decorin glycans were found to be more complicated, but also had a relatively small number of modification patterns. An example is presented in Figure 11, which shows the GAG chain of decorin connected to the protein core, a representative CID spectrum of a dp20 GAG, and the overall sequence motif for the GAG chain. Although top-down analysis of the full-length glycans from two PGs has been reported, it is unlikely that the top-down strategy will be useful for other PGs. Biologically relevant PGs are likely to rely on the bottom-up methods described in this review. Structure determination of GAGs can provide valuable insight into the impact of modification patterns for GAG–protein binding to answer a variety of biological issues.

## Conclusions

While challenges remain for the analysis of GAGs, recent advances and research in MS of complex GAGs has paved the way for faster and more complete analysis. The evolution of MS/MS methods has led to more detailed structural characterization for this class of carbohydrates. Structures of GAG chains of different lengths and modifications can be determined by MS/MS, especially when using electron-based methods. Recent advances in GAG analysis software have led to a faster analysis process and a simplified way to identify unknown sample structures. With the wide variety of separation techniques that can be coupled to MS, more complex samples can be explored on a reasonable timescale to determine composition and sequence information. GAG analysis has mostly focused on shorter chains, but the sequencing of intact GAG chains such as bikunin and decorin demonstrates the capabilities of MS analysis. Future developments will integrate the isolation of biologically relevant regions of GAG chains with MS analysis, to address significant problem in biology and medicine.

## Conflicts of interest

The authors declare no competing interests.
